# Unusually High Temperature in a Case of Sunstroke

**Published:** 1878-10

**Authors:** Chas. T. Parkes


*Unusually High Temperature in a Case of Sunstroke.*

At 3 p, m. on the 17th day of July I was called to see P. H——, a brick-yard employé. I found the man entirely unconscious, face flushed, breathing laboriously, with now and then slight convulsive contraction of the muscles of mastication, and skin dry and excessively hot—so hot that to touch him was unpleasant. On inquiry I ascertained that he had not been feeling very well all day, and about half an hour before my arrival fell down unconscious, and had been carried under the shed where I found him.

Astonished at the great heat of his skin, I determined to ascertain his temperature before carrying out the treatment I had concluded upon. While removing his clothes to get at the axilla, etc., I despatched some bystanders for a large cake of ice. I placed the thermometer in his axilla—first noticing that the mercury stood at 95°—and drew his arm firmly against the side of the body. The mercury began to rise rapidly, and in less than a minute reached the very top of the tube in the thermometer. Of course I was amazed, but there was no doubting the fact of the register. My thermometer is of the best English make, and has been proven trustworthy. It is scaled to 112°, the tube reaches a distance equal to 1° above, so that the case had a temperature marked to 113°. The rapidity with which the mercury rose to this height convinces me that the register would have been higher had there been more space for its markings. I entertained very little hope of the man's recovery. While waiting for the ice, I uncovered his body of clothing and dashed cold water all over him repeatedly, with, at first, scarcely any response from his nervous system. Soon, however, there followed a deeper and more complete respiration after each application of water. When the ice arrived I placed large pieces over the groins, in both axillae, to the sides of the neck, and embanked the head in small fragments, covered the abdomen, and in fact put it everywhere that it could be made to hold, choosing by preference such parts of the body where large blood-vessels come near to the external surface. By this means—ice and cold water—in an hour's time the temperature was reduced to 99½°. As the temperature came to 100° the man began to show more response to irritation of any kind—he began to vomit and ejected large quantities of yellow-colored fluid. Still he showed no signs of returning consciousness other than low muttering and response to irritation, such as sprinkling water on his face. After keeping the man's temperature below 100° for half an hour, I sent him home, with instructions to have one teaspoonful of Hoffman's anodyne administered to him every hour until I saw him again.

I saw him in two hours; he was still semi-unconscious and vomiting watery mucus, yellow in color. His temperature had risen to 103°. I reapplied ice and water until he was cooled off, and ordered the anodyne continued, together with beef tea, or soups of any kind. I visited the man the next morning, found him talking cheerfully to his friends, and complaining only of headache and a little soreness of the limbs—temperature 100°. The man convalesced rapidly. CHAS. T. PARKES.

